# Antibacterial and anti-*Trichomonas Vaginalis* effects of *Rosa Damascena* mill petal oil (a persian medicine product), aqueous and hydroalcoholic extracts

**DOI:** 10.1186/s12906-021-03434-8

**Published:** 2021-10-20

**Authors:** Fatemeh Saghafi, Farzaneh Mirzaie, Elham Gorji, Razieh Nabimeybodi, Mehdi Fattahi, Hamed Mahmoodian, Rahele Zareshahi

**Affiliations:** 1 Department of Clinical Pharmacy, Faculty of Pharmacy and Pharmaceutical Sciences Research Center, Shahid Sadoughi University of Medical Sciences, Yazd, Iran; 2 Department of Parasitology and Mycology, School of Medicine, Shahid Sadoughi University of Medical Sciences, Yazd, Iran; 3 Herbal Medicines Research Center, Student Research Committee, School of Pharmacy, Shahid Sadoughi University of Medical Science Health Services, Yazd, Iran; 4 Department of Persian Medicine, School of Persian Medicine, Shahid Sadoughi University of Medical Sciences, Ardakan, Yazd, Iran; 5 Department of Microbiology, School of Medicine, Shahid Sadoughi University of Medical Sciences, Yazd, Iran; 6 Management Department, Meybod University, Meybod, Iran; 7 Department of Pharmacognosy, School of Pharmacy, Shahid Sadoughi University of Medical Sciences, Yazd, Iran

**Keywords:** Antibacterial activity, MIC, *Rosa damascena*, *Trichomonas vaginalis*

## Abstract

**Background:**

Oils in traditional medicine are important products and used routinely for therapeutic purposes. Rose oil (*Rosa damascene* Mill), a product of Persian medicine, is advised for the treatment of Infectious diseases related to the female genitourinary tract. In the present study*, R. damascena* petal oil, aqueous, and hydroalcoholic extracts were evaluated for their *in vitro* antibacterial and anti-*Trichomonas vaginalis* effects.

**Methods:**

Anti-*trichomonas* activity evaluation of extracts and oil were assayed by the Homocytometery method. Their antibacterial effects against *Escherichia coli*, methicillin-resistant *Staphylococcus aureus*, *Pseudomonas aeruginosa*, and clinically isolated Group B *Streptococcus* were assayed by broth microdilution in 96-well plates.

**Results:**

The MIC of hydroalcoholic and aqueous extracts ranged from 25-50 and 25-100 mg/ml, respectively. Rose oil at all administered doses failed to show any antibacterial activity.

**Conclusion:**

All extracts and oil concentrations showed some degree of growth inhibition activity on *T. vaginalis*; however, hydroalcoholic extract was more efficient.

## Introduction

Infectious diseases represent a serious health problem today and account for one-third of all deaths worldwide. Antimicrobials of plant origin have enormous therapeutic potential, as they are effective in the treatment of infectious diseases while simultaneously mitigating many of the side effects often associated with synthetic antimicrobials [1]. Resistance to antimicrobial and anti-parasitic drugs has become a global challenge due to inappropriate and excessive consumption of these drugs. Bacteria and viruses developed their resistance mechanisms like enzymatic degradation, target alteration, decreased uptake and over expression of efflux pump proteins, every day and synthetic chemicals used for microorganism controls have some limitations like carcinogenic effects, their toxicity and, environmental risks [2–5].

At present, traditional medicine is more acceptable than in the past. It is cheaper and more accessible than conventional treatment [6]. World Health Organization (WHO) supports traditional medicine because traditional literatures are source of treatment strategies for different people in the world [7]. Iranian traditional medicine (Persian medicine) has deep roots in the history of medicine. Some Persian literatures, such as the Canon of Avicenna (Iranian scientist) and Liber Continent of Rhazes (Iranian scientist), was used as the main reference books in western universities until the 17th century AD [8]. Most current ethnopharmacological knowledge in Iran has been derived from historical manuscripts. Pharmaceutical manuscripts of Persian scholars contain various therapeutic dosage forms and treatment strategies. Over 100 dosage forms have been introduced in pharmaceutical literatures of Persian medicine but they need updates and quality controls [9, 10].

Oils are one of the oldest forms of natural medicine in the world. Traditional Chinese, Indian, and Egyptian medicines use oils for medical purposes. In Persian medicine (PM), too, oils are important products and used routinely for therapeutic purpose s[10]. Rose oil is advised for the treatment of a Qorohe-rahem (Uterus), an infectious disease of the uterus. Signs of this disease include cervicitis [11]. Cervicitis is inflammation of cervix caused by some microorganisms colonization [12]. *Pseudomonas aeruginosa*, *Escherichia coli*, Methicillin-resistant *Staphylococcus aureus* (MRSA), Group B *Streptococcus* (GBS), and *Trichomonas vaginalis* are the microorganism, which found in vagin of women with cervicitis [12–14]. Trichomoniasis, the most prevalent non-viral sexual infection with roughly 156 million newly infected people worldwide per year, has been related to various discomforts such as pre-term delivery, high infant mortality, and low birth weight, and it makes patients more susceptible to HIV infection. It effects women more than men [15]. The most common treatment of trichomoniasis is Metronidazole (MTZ) or Tinidazole, antibiotics and members of the 5-nitroimidazole family, which are one of the most important agents for the treatment of anaerobic infections [3]. Yet, these drugs have many side effects, particularly when higher doses are needed in steadily increasing resistant cases. Metronidazole causes nausea and dizziness, induces hypersensitivity reactions and dermatological symptoms, and has teratogenic and carcinogenic effects on the fetus. Currently, no alternative safe therapy is approved to overcome the severe side effects of the traditional drugs or to treat refractory cases of trichomoniasis. These facts emphasize the need for other safe, effective treatment modalities [16].

In PM Rose oil is recommended for uterus diseases. *Rosa damascene* Mill of the Rosaceae family and have several pharmacological properties including anti-HIV, antibacterial, antioxidant, antitussive, hypnotic, antidiabetic. *R. damascena* originated in Iran and then brought to Europe and cultivated there [17].

The present study was designed to evaluate the antimicrobial effects of rose oil and hydroalcoholic extract (HAE) and aqueous extract (AE) against some microorganisms that cause infections of the uterus, especially cervicitis, like *P. aeruginosa*, *E. coli*, MRSA, GBS, and *T. vaginalis* [12–14].

## Materials and methods

In this experimental study, plants of *R. damascena* were collected from Kashan province, Iran. Species identification and authentication were achieved in the Botany Department of Shahid Sadoughi University of Medical Sciences, Yazd, Iran, and a plant specimen was deposited there with voucher number ssu.6769. The study protocol complied with relevant institutional, national, and international guidelines and legislation.

### Chemicals and materials

The *T.vaginalis* culture was TYIS-33. Olive oil was purchased from Famila Company. Metronidazole, Ethanol, Tween 80, KH_2_PO_4_, K_2_HPO_4_, Folin Ciocalteu, Gallic acid, NAHCO_3_, Ascorbic Acid, Cyctein Hydrochloride, Ferric Amonium Citrat, NaCl, Glucose were purchased from Merck company. Tripan Blue, MTT, PBS tablet were purchased from Sigma Aldrich. Yeast extract cultures, Triptocase were purchase from Qlab, Canada.

### Preparation of extracts

Hydroalcoholic extract was prepared using the maceration method. *R. damascenea* petals were ground into a fine powder and macerated individually at a ratio of 10 g of ground plant material in 100 ml of 80% (v/v) ethanol for 72 hours. Extraction was done at room temperature under occasional shaking by a magnetic stirrer. Then, the solution was purified by Buchner funnel and concentrated [18]. Aqueous extract was prepared by the diffusion method. *R. damascenea* petals were ground into a fine powder and heated in water (80°C) for 30 min. Then the solution was filtered by Buchner funnel and concentrated [18].

Prepared extracts were stored at 4 °C until further use.

### Preparation of rose oil

Rose oil was prepared according to the methodology of Persian medicine called “Qarabadin”. Fifty g of plant powder was soaked in 300 mL distilled water overnight and then heated to 80 °C on a heater for one h. The solution was then filtered using the vacuum Buchner funnel. The rose extract was mixed with an equal amount of commercial olive oil (manufactured by Oila). The extract was heated again for complete evaporation of aqueous extract, leaving only the oil [19]. The prepared oil was stored in a dark bottle in the absence of light until required [19].

### Total phenolic content

The total phenolic content of extracts and oil was determined by the Folin–Ciocalteu method. Gallic acid was used as a standard, and total phenolics were expressed as mg/g of gallic acid equivalents (GAE). To prepare the oil extract, equal volumes of oil and methanol were mixed and centrifuged at 4000 rpm for 13 minutes. Then the methanolic extract of the oil was isolated and used for standardization. This was repeated three times. Concentrations of 10, 20, 40, 60, 80, 100, and 200 μg/ml of gallic acid were prepared. 0.1 ml of each sample were introduced into test tubes and mixed with 0.5 ml of a 10-fold diluted Folin-Ciocalteu reagent and 0.4 ml of 7.5% sodium carbonate after 3-8 minutes. The tubes were covered with Parafilm and allowed to stand for 30 min at room temperature before the absorbance was read at 760 nm spectrophotometrically. All determinations were performed in triplicate. Total phenolic content was determined as mg of gallic acid equivalent per gram using the equation obtained from a standard gallic acid calibration curve [20].

### Secondary metabolite tests

Tests to identify flavonoids, alkaloids, anthraquinons, and tannins were performed as described previously [21].

### Flavonoid identification

To 1 cc of the extract, 0.5 g of zinc or magnesium powder and 2 drops of 2 N hydrochloric acid were added; after one minute, 10 drops of concentrated hydrochloric acid were added to the test solution. A cherry-red color developing would indicate a positive test [21].

### Tannin identification

A few drops of hydrochloric solution (6.7 g per 100 ml of water) were added to 10 ml of the extract; the development of a blue or green color indicated the presence of tannins [21].

### Anthraquinone identification

Borntraeger-Reaction test:

Five ml of 2 N sulfuric acid was added to 2 ml of extract for a short time and then dried by 10 ml of toluene in a decanter. The solution was decanted, the toluene phase was separated, and the solution was smoothed. The presence of anthraquinone compounds, the solution would turn yellow. This phase was then decanted with 2 ml of normal NaOH. After separation, the aqueous phase was completely red, and the other phase was colorless [21].

### Alkaloid Identification

Twenty cc of the extract was heated by 1 cc double normal hydrochloric acid and 9 cc distilled water for 2 to 5 minutes in a bain-marie water bath. After cooling, a few drops of Dragendorf reagent were added to a watch glass, and discoloration and sedimentation were examined [21].

### Preparation of the bacterial strains

The commercial strains (*Pseudomonas aeruginosa* ATCC 27853, *Escherichia coli* ATCC 25922, and methicillin-resistant *Staphylococcus aureus* ATCC 33592) and clinically isolated strains (*Streptococcus* B) causing infections were provided by the Laboratory of Industrial Microbiology, Shahid Sadoughi University of Medical Sciences, Yazd, Iran.

Stock bacterium cultures were maintained at room temperature for 2 h. Each strain was streaked on nutrient and blood agar plates and incubated at 37 °C for 24 h. The inoculum was prepared by emulsifying a minimum of three colonies from those plates in sterile 0.9% NaCl (w/v) until 10^8^ CFU per ml (0.5 McFarland scale) were formed. The use of laminar hood equipment assured the sterile conditions of the procedures [22].

### Preparation of *Trichomonas Vaginalis*

*T. vaginalis* strains were isolated from the vaginal discharge of women with *Trichomonas* vaginitis who referred to the healthcare centers of Yazd, Iran, transferred to TYI-S-33 culture medium, and kept in the Parasitology Research Laboratory of Shahid Sadoughi University of Medical Sciences until use. All methods were carried out in accordance with relevant guidelines and regulations or Declaration of Helsinki. *T. vaginalis* cells were collected from the logarithmic growth phase and their count numbers were estimated using a hemocytometer slide. Finally, a count number of 1×10^5^/ml cells were used for the anti-*T. vaginalis* effects of *R. damascena.*

### Sterilization

The extract and oil solutions were sterilized by filtration on 0.45 μm filters.

## Antibacterial effects

### MIC (minimal inhibitory concentration) determinations

MIC tests were performed using the broth microdilution method. Assays were carried out in Mueller Hinton broth (MH), as recommended by the National Committee for Clinical Laboratory Standards (NCCLS 1999b). Serial doubling dilutions of plant extracts in MH broth (ranging from 0.195-100 mg/ml) and plant oil in MH broth supplemented with Tween-80 (at a final concentration of 100 μL per ml) were prepared. Each dilution (100 μL) was dispensed into the wells, then inoculated with 100 μL of the bacterial suspension and mixed thoroughly. For each experiment, negative (growth) and positive (sterility) controls were used [22].

For hydroalcoholic, aqueous extracts, and rose oil, bacterial growth controls were made by replacing extracts and oils with the same volume of 80% ethanol, MH broth, and Tween-80, respectively, to eliminate the possible antibacterial effect of the solvents. Sterility controls were prepared using MH broth medium alone. The final volumes in wells were 200 μl. The plates were covered with a sterile plate sealer and incubated at 37 °C for 24 h. After incubation, 10 μl of 5% 3-(4,5-dimethylthiazol-2-yl)-2,5-diphenyl tetrazolium bromide (MTT) was added to each well; the plate was then reincubated at 37 °C for 30 minutes. Bacterial growth was determined by observing the change of color MTT in the microplate wells (pinkish-red formazan when there was growth, and clear solution when there was no growth) [23]. MIC was defined as the lowest sample concentration showing no color change (clear) and exhibited complete bacterial growth inhibition [22, 24].

### MBC (minimal bactericidal concentration)

To determine the MBC, 10 μl of broth aliquots were taken from each well with an extract concentration equal to or higher than the MIC values and incubated in MH agar at 37 °C for 24 h. MBC was determined as the lowest concentration that showed no bacterial growth in the subcultures. Each experiment was performed in triplicate [25].

### *In vitro* anti-Trichomonal assay

To evaluate the anti-Trichomonal effects of *R. damascena*, extracts and oil, extract concentrations of 0.0195 - 10 mg/ml, and oil concentrations of 0.039 - 20 mg/ml were disposed in phosphate buffer saline (PBS) and added to the microtubes. Metronidazole (64 μg/ml) and PBS were used as positive and negative controls, respectively. Then 100 μl of medium containing about 100 live *T. vaginalis* microorganisms were added to each tube. All tubes were incubated at 37 °C and the number of live parasites in each tube was counted following 24, 48, and 72 hours of incubation. For every sample and each time, a tube was first shacked, and then live cells were counted using a hemocytometer slide. The active parasites and those with moving flagellum were considered alive. Each experiment was performed in triplicate. The number of live parasites was compared with the positive and negative controls. The growth inhibitory percentage (GI%) was calculated and reported using the following formula:

$$\mathrm{GI}\%=\mathrm{a}-\mathrm{b}/\mathrm{a}\times 100.$$

In the formula:

- Stands for an average of live parasites in the negative control tube, and (b) stands for the average of live parasite count in the test tube [26].

### Statistical analysis

Data was computerized and statistically analyzed using SPSS version 25.0 and one-way ANOVA.

## Results

### Total phenol content determination

Based on the absorbance values of the extract solutions, the colorimetric analysis of the total phenolics of the extracts was performed and the results compared with those of the standard solution of gallic acid equivalents. Total phenolic contents of hydroalcoholic extract, aqueous extract, and oil extract was 40.47 mg GAC/g, 42.2 mg GAC/g, and 26.18 mg GAC/g, respectively.

As reported in Table 1, between the secondary metabolites, alkaloid was not found in any extracts and test for anthraquinones identification in oil was negative but Tannins and Flavonoids were found in oil and, extracts.

**Table 1:** Results of Flavonoid, Alkaloid, Anthraquinone and, Tannin tests identification in HAE, AE and, oil of *R.damascena*

Type of Extract	Tannin	Alkaloid	Anthraquinone	Flavonoid
AE	**+**	**-**	**+**	**+**
HAE	**+**	**-**	**+**	**+**
Oil	**+**	**-**	**-**	**+**

*HAE* Hydroalcoholic extract, *AE* Aqueous extract, *R.damascena Rosa damascena*

### Antibacterial activity

MIC values are summarized in Table 2. The MIC of hydroalcoholic and aqueous extracts ranged from 25-50 and 25-100 mg/ml, respectively, and rose oil did not present any antibacterial activity against the strains tested. The MIC of the aqueous extract included 25, 25, 25, and 50 mg/ml for *GBS*, *E. coli*, MRSA, and *P. aeruginosa,* respectively.

**Table 2:** MIC values of *R. damascena* extracts by micro broth dilution assay

Type of microorganism	Hydroalcoholic (mg/ml)	Aqueous (mg/ml)
	MIC	MIC
MRSA	25	25
*Streptococcus* B	25	25
*E. coli*	50	25
*P*. *aeruginosa*	100	50

*MIC* Minimum inhibitory concentration, *MRSA* Methicillin-resistant *Staphylococcus aureus*, *E. coli Escherichia coli, P. aeruginosa Pseudomonas aeruginosa*

### Anti-*T. vaginalis* effect

**Fig. 1:**
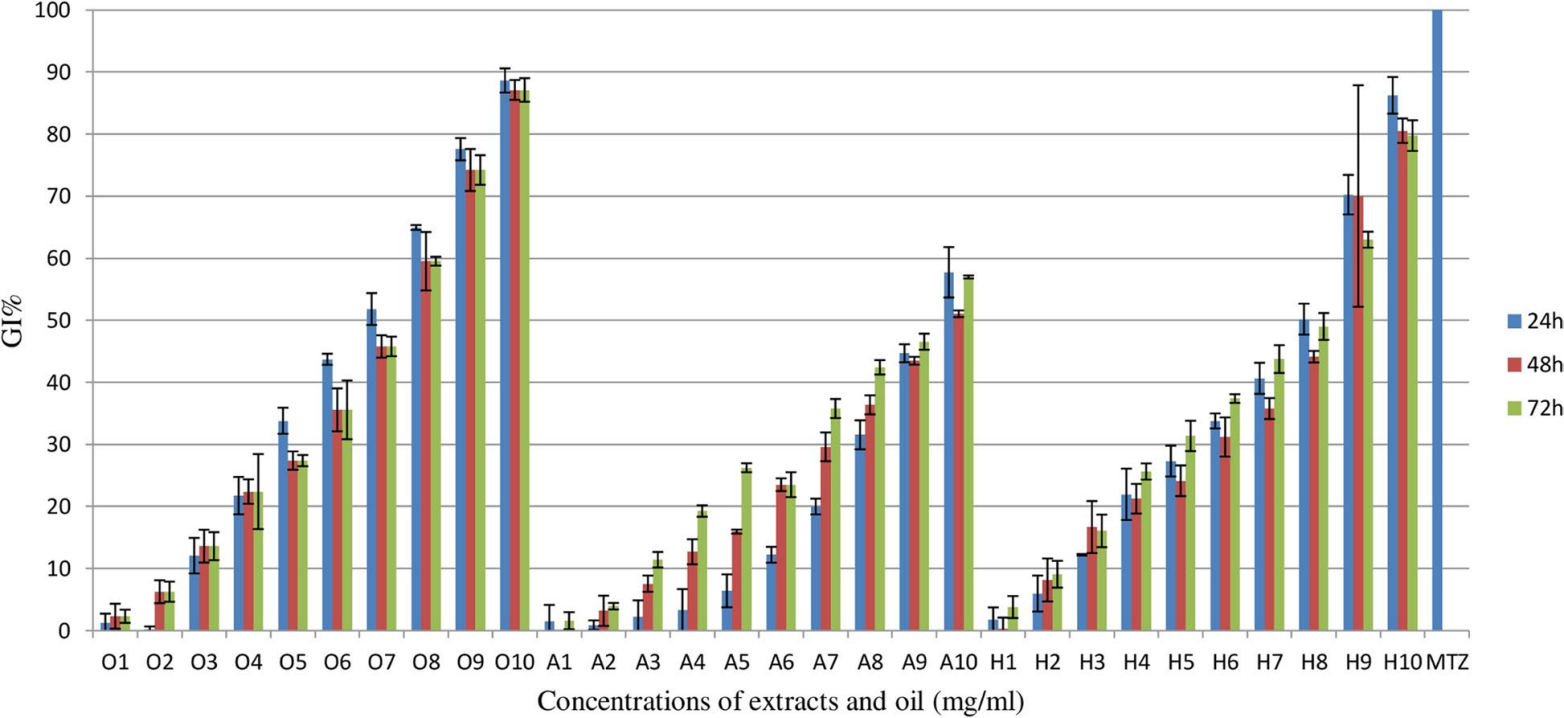
The inhibitory effects of *R. damascena* oil (O1-O10: 0.039 - 20 mg/ml), Aqueuse extract (A1-A10: .0195 - 10 mg/ml), Hydroalcoholic extract (H1-H10), and Metronidazole (0.064mg/ml) on *T. vaginalis* growth in different concentrations and incubation times. GI%: percentage of growth inhibitory of parasit. 24h, 48h and 72h were the times that GI calculated

The results of anti-*T. vaginalis* activity of rose oil and extracts are shown in Fig. 1 and the results of half maximal inhibitory concentration (IC50) shown in Table 3. As shown in Table 3, IC50 of rose oil after 24, 48, and 72 h were 1.79, 2.24, and 6.11 mg/ml, respectively and there is not any significant different (*P*>0.05). IC50 hydro alcoholic extract were 1.41, 1.84, and 1.44mg/ml after 24, 48, and 72 h. IC50 aqueous extract was 15.24, 12.64, and 5.56 mg/ml after 24, 48, and 72h. In addition, the difference between IC50 of extracts in different days were not statistically significant (*P*>0.05).

**Table 3:** The IC50 values determined for oil and extracts of *R. damascena* against *T. vaginalis*

Materials	IC50%(mg/ml)			***P***-value
	24h	48h	72h	
Oil	1.79	2.24	6.11	>0.05
Hydroalcoholic extract	1.41	1.84	1.44	>0.05
Aqueous extract	15.24	12.64	5.56	>0.05
*P*-value	>0.05	<0.05	>0.05	

*IC50* half maximal inhibitory concentration, *R.damascena Rosa damascene*, *T.vaginalis Trichomunas vaginalis*

The maximum GI% of oil was 88.62, 87.10, and 71.27% in 24, 48, and 72h, while the maximum GI% of the HA extract was 86.23, 80.54, and 79.75% and of AE was 57.73, 51.04, and 56.96%, respectively.

**Fig. 2:**
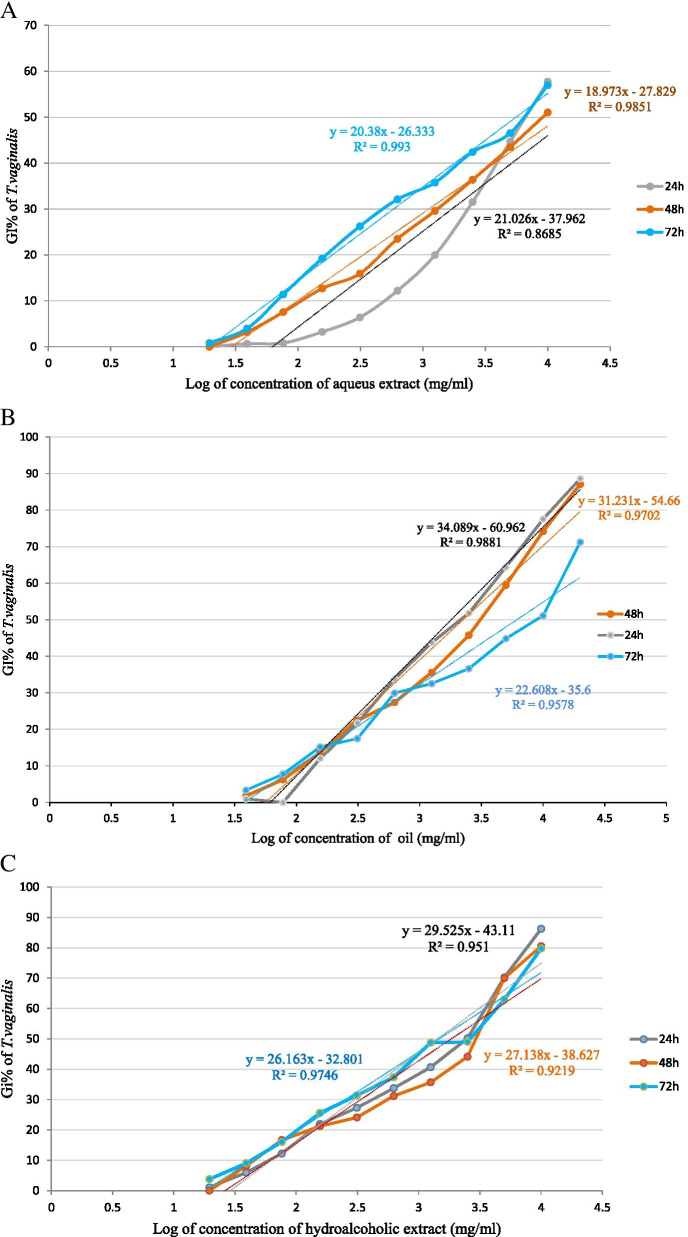
GI% of *T. vaginalis* vs. logarithm (Log) of concentration of rose oil (**A**), aqueous extract (**B**), and hydroalcoholic extract (**C**) in three times, 24, 48 and,72h. GI%: percentage of growth inhibitory of parasit

The results of comparing the percentage of growth inhibitory *T. vaginalis* are shown in Fig. 2.

In our experiments, no trophozoite was found in the reference tubes at a Metronidazole concentration of 64 μg/ml.

## Discussion

In the present study, the effects of *R. damascena* extracts and oil on four species of bacteria and *T. vaginalis* growth which have role in infectious uterine diseases were evaluated [12–14].

The results of this study show that all bacteria were resistant to rose oil. The HAE had the greatest effect in inhibiting the growth of all bacteria and its MIC for GBS, MRSA, *P. aeruginosa* and *E.coli* was 25, 25, 50 and 25 mg/ml, respectively.

*E. coli* is one of the most common organisms found in the genital tract of 9-28% of non-pregnant women and 31-34% of pregnant women. *E coli* infection during pregnancy can cause urinary tract infections, intra-amniotic and puerperal infections through fecal-vaginal urinary / neonatal transmission. Main choice for obstetric and neonatal infection is Ampicillin but Ampicillin resistance among *E. coli* strains is increasing [27]. Results of this study show that AE was more sensitive than HAE against *E.coli*. The results of Hirulkat et al. confirm our results [28]. Tannins and flavonoids have an important role in antimicrobial activity of herbal extracts. Some studies reported antimicrobial activity of flavonoids and tannins particularly against *E.coli* [29, 30]*.*

Another important bacterium in vaginal infections is GBS. GBS is generally asymptomatic and is colonized as a member of the vaginal or gastrointestinal microbiota. Nevertheless, it can cause life-threatening infections in infants and increasingly in adults. Maternal colonization is the most important risk factor for neonatal GBS infection and it is estimated that rectovaginal colonization is 12-36% in the worldwide population [31]. In this study HAE and AE had the same effect and in 25mg/ml shown inhibitory effect. Mahboubi et al reported the MIC of Rose essential oil is 1μg/ml. the cause of this difference is the difference between composition of extract and essential oil. The antimicrobial effect of rose oil is related to geraniol, ß-citronellol and, eugenol [32]. The main components in extracts are phenolic component. The antimicrobial effect of phenolic compounds depends on amount of them on extracts. At low concentrations they are able to interfere with enzymes involved in the production of energy and at higher concentrations, they can induce the denaturation of proteins [33]. Kaempferol, cyanidin3,5, D-glycoside, quercetin, and gallic acid are the major active phenolic compounds in *R.damascena* [34].

*P. aeruginosa* is one of the most common causes of infection in humans and warm-blooded animals, urinary tract infection and mastitis. This pathogen is one of the most important problems for clinicians both in the community and in the hospital because it has led to an increase in mortality due to drug resistance. In this study HAE an AE inhibited *P.aeruginosa* in 100mg/ml and 50 mg/ml respectively. Among microorganisms in this study, *P.auroginosa* was less sensitive. *P. aeuroginosa* by different mechanism have resistance against antiobiotic treatments like ß-lactams, quinolones, aminoglycosides, and colistin, like Interactivity enzymes, target alterations, efflux pumps, porin deficiency and, adaptive mechanisms [35].

In the current study, aqueous and hydroalcoholic extracts showed inhibitory effects on gram-positive bacteria in lower concentrations than on gram-negative bacteria. These results were supported by other studies. Trong et al. have been reported *P. timera* essential oil inhibited Gram positive more than Gram negative bacteries [36]. Shohayeb M et al. studied the antimicrobial and antifungal effects of *R. damascena* and showed that gram-positive bacteria had a higher susceptibility than gram-negative [37]. Gram negative bacteria’s have more complex cell wall than Gram-positive bacteries so extracts can penetrate to Gram-positive bacteria [2]. Complex cell wall in Gram negative bacteries contains asymmetric bilyer of phospholipid, penicillin-binding proteins and, porins which are the selective barrier to penetrating or bypassing of antibiotic molecules to cell wall and antibiotic could act on target cells. Nevertheless, any changes in this cell wall structure can changes susceptibility of antibiotics [35].

In this study HAE and AE inhibited MRSA in the same concentration (25mg/ml). but in study of Thomas et al aqueous extract of *R.damascena* was more potent than ethanolic extract against *S.aureus* and they weren’t reported any antimicrobial effect against *E.coli*. It is maybe because of the difference between methods of extraction. In this study maceration method was used for extraction and in Thomas’s study Soxhlet method. In Soxhlet extraction because of heating maybe some constituents decomposed but in maceration there is not any heating [38].

The purpose of the microdilution method is to evaluate MIC and to determine the lowest concentration of an antibacterial agent necessary to inhibit the growth of a microorganism. The MIC and MBC results revealed the nature of the antimicrobial activity, whether it was bacteriostatic or bactericidal. The evaluation of the MBC revealed that extracts did not show any bactericidal activity against strains tested. Bassam Abu-Shanab et al. investigated the antibacterial effects of aqueous and ethanolic extracts of *R. damascena* on MRSA. The results of their study indicated the bacteriostatic and bactericidal effects of rose extracts on this resistant hospital microorganism, but no bactericidal effects were observed in the current study [39]. Mankar S studied the antibacterial activity of different species of rose against pathogenic bacteria and reported that 19 species of rose showed moderate to strong effects and three species showed weak antimicrobial effects [40].

Oil has not shown any antibacterial effect. Probably, the solubility of oil in the medium was not good. The flavonoid test for oil extract was positive, so the oil did contain flavonoids for an antibacterial effect, but they probably could not be released in the bacterial medium.

Oils in PM are prepared by boiling aqueous extract in sesame oil, so heating can cause the loss of phenols, so the total phenol test for oil has given the lowest amount. However, it showed significant effects in eliminating *Trichomonas*, therefore other factors besides phenolic compounds also play a role in this effectiveness. Studies showed essential oils by increasing penetration of cell membrane causes ions and molecules have been exited from cells and made cell apopthosis. However, rose oil as a Persian medicine product and a fixed oil is hydrophob so probably treat like essential oils [2].

Medicinal oils have been used in PM for thousands of years to treat various disorders. Oils in PM have been used topically, oral and even nasal and vaginal. There are several modern studies about different effects of oils as a Persian medicine product, such as anti-inflammatory and neuroprotective, antiepileptogenic and neuroprotective of some these medicinal oils, which can be considered as evidence of these medicinal oils effectiveness. One of methods for preparing medicinal oils, which also used in this study, is boiling and evaporation method. In this method the aqueous phase becomes trapped in the oil phase following evaporation. However heat-sensitive component may deleted by overheating but many useful effects for these oils have been reported via current pharmacological studies [10].

Topical application of rose oil in PM is recommended for the treatment of wounds, burning, and uterine diseases, especially Qoruh-e-rahem which is a disease like cervicitis in conventional medicine [11, 41]. Anti-inflammatory and antimicrobial effects are important for new drug for treatment of Cervicitis as a disease which is complex of inflammation and infection [42]. Different studies have been done on the extracts of various parts of *Rosa damascena*. Studies performed by Rakhshandeh et al. reported the hypnotic and analgesic effects of ethanolic extract of *Rosa damascena* shown in animals [43]. Hajhashemi et al. showed the analgesic and anti-inflammatory effects of hydroalcoholic extract of *Rosa damascena* in mice [44]. Latifi et al. have been reported anti-inflammatory effect of Hydroalcoholic extract of *R. Damascena* in acid acetic colitis ant its antioxidant activity [45]. Given that cervicitis is an inflammatory and infectious disease, rose oil through its anti-inflammatory and anti-*Trichomonas* effect may be effective against this disease.

Antibiotics are currently used to treat infectious diseases, but drug resistance is one of the biggest problems worldwide, killing many people over the past decade. Every day, bacteria and viruses improve their resistance mechanisms. On the other hand, the use of synthetic chemicals to control microorganisms has many limitations, including carcinogenic effects, acute cystitis, and environmental hazards. Therefore, it is necessary to produce new antibiotics with high and non-toxic effusion [2].

In this study, the *in vitro* anti-*T. vaginalis* activities of *R. damascena* oil and extracts against *T. vaginalis* trophozoites were assessed by Trypan Blue exclusion assay. The results showed that all the tested extracts were effective in inhibiting the growth of *T. vaginalis* trophozoites in a dose-dependent manner after 24, 48, and 72 h of incubation. Moreover, the HAE was more effective, as it demonstrated lower IC50 values for trophozoites of *T. vaginalis*.

Given that the constituent of essential oils and extracts of different plants under particular biotic/abiotic stress conditions are different so the effects of them on microorganism would be different [46]. While in this study the maximum GI% of oil extract was 88.62%, the maximum GI% of the HA extract was 86.23%, and of queues extract was 57.73% in 24 h; Ezatpour et al. reported that the *Lavandula angustifolia* essential oil in 0.1% of concentration killed all live *T. vaginalis* cells in 90 minutes [47]. Alyasari et al. stated that the growth rate, viability, and motility of *T. vaginalis* were inhibited 20% after 24h entirely when using the aqueous garlic extract [48].

Tests for tannin and flavonoid identification for extracts and oil in this survey were positive. Different studies have been reported flavonoids and condensed tannin have shown anti-*Trichomonas* activity. Tasca et al. reported that in the *Quillaja*, *Passiflora*, and *Ilex* species, saponins exhibited anti-*Trichomonas* activity [49]. In the current study, none of the extracts had alkaloids, so it cannot be said that alkaloids showed anti-*Trichomonas* activity; however, there were tannins in three extracts, so the tannins may be responsible for this effect. Tannins are water-soluble polyphenolic compounds possessing variable molecular weights that are widely found in nature and abundantly in fruits, vegetables, and other foods. Silva et al. reported the anti-*Trichomonas* activity of *Poincianella (Caesalpinia) microphylla* fractions. They reported that the main compounds identified were hydrolyzable tannins (gallitannins and ellagitannis), such as *O*-digalloyl hexoside, *O*digalloyl HHDP-hexoside, Tri-*O*-galloyl HHDP-hexoside, *O*-galloyl HHDP-DHHDPhexoside, and their isomers. The first structural description of anti-*T. vaginalis* condensed tannins activity was provided by Patrícia de Brum Vieira et al. [50].

Further studies are required for the isolation and purification of the active ingredients of *R. damascena* responsible for these inhibitory effects for the tested bacteria and *T. vaginalis* to better understand the mechanism of such actions.

## Conclusion

The main purpose of this study was to determine if rose oil as a Persian medicine product can be effective against inflectional disease, as reported in PM references. The HAE extract showed a better effect against bacteria, and the oil showed the best effect against *Trichomonas*. Thus, rose oil as a Persian medicine product could be a candidate medicine for the treatment of Trichomonasis, but it must be studied further. The anti-*Trichomonas* activity of this oil is one of its mechanisms in uterine infection diseases.

## Data Availability

The datasets used and analyzed during the current study are available from the corresponding author on reasonable request.

## Abbreviations

MTZ: Metronidazole
PM: Persian Medicine
GAE: Gallic Acid Equivalents
CFU: Colony Forming Units
MH: Mueller Hinton
MTT: 3-(4,5-dimethylthiazol-2-yl)-2,5-diphenyl tetrazolium bromide
GI: Growth Inhibitory
MBC: Minimal Bactericidal Concentration
MIC: Minimal Inhibitory Concentration
PBS: Phosphate Buffer Saline
IC50: Inhibitory Concentration
MRSA: Methicillin-Resistant Staphylococcus Aureus

## References

[1] Perumal S, Pillai S, Cai LW (2012). Determination of minimum inhibitory concentration of euphorbia hirta (L.) extracts by tetrazolium microplate assay. J Nat Prod.

[2] Donadu MG, Trong Le N, Viet Ho D, et al. Phytochemical compositions and biological activities of essential oils from the leaves, rhizomes and whole plant of hornstedtia Bella Škorničk. Antibiotics. 2020;9(6). 10.3390/antibiotics9060334. 10.3390/antibiotics9060334 PMC7344524 32570731

[3] Gajdács M, Spengler G, Urbán E. Identification and antimicrobial susceptibility testing of anaerobic bacteria: Rubik’s cube of clinical microbiology? Antibiotics. 2017;6(4). 10.3390/antibiotics6040025. 10.3390/antibiotics6040025 PMC5745468 29112122

[4] Amanpour R, Abbasi-Maleki S, Neyriz-Naghadehi M (2015). Antibacterial effects of solanum tuberosum peel ethanol extract in vitro. J Herb Med Pharmacol.

[5] Gajdács M (2019). The concept of an ideal antibiotic: implications for drug design. Molecules..

[6] Hashempur MH, Lari ZN, Ghoreishi PS (2015). A pilot randomized double-blind placebo-controlled trial on topical chamomile (Matricaria Chamomilla L.) oil for severe carpal tunnel syndrome. Complement Ther Clin Pract.

[7] Pedreira-Robles G, Vasco-Gómez A, Martínez-Delgado Y (2020). Traditional and complementary medicine in a nephrology department: practitioner knowledge and advice. Br J Nurs.

[8] Larijani B, Esfahani MM, Moghimi M (2016). Prevention and treatment of flatulence from a traditional Persian medicine perspective. Iran Red Crescent Med J.

[9] Zarshenas MM, Zargaran A, Müller J (2013). Nasal drug delivery in traditional Persian medicine. Jundishapur J Nat Pharm Prod.

[10] Hamedi A, Zarshenas MM, Sohrabpour M (2013). Herbal medicinal oils in traditional Persian medicine. Pharm Biol.

[11] Nabimeybodi R, Zareshahi R, Tansaz M (2019). Scientific evaluation of medicinal plants used for the treatment of cervicitis (Qorohe- Rahem) in Iranian traditional medicine. Iran J Pharm Res.

[12] Ortiz-de la Tabla V, Gutiérrez F (2019). Cervicitis: etiology, diagnosis and treatment. Enferm Infecc Microbiol Clin.

[13] Deng L, Schilcher K, Burcham LR, et al. Identification of key determinants of staphylococcus aureus vaginal colonization. mBio. 2019;10(6). 10.1128/mBio.02321-19. 10.1128/mBio.02321-19 PMC6935855 31874913

[14] Vander H, Prabha V (2019). Colonization of mouse vagina with pseudomonas aeruginosa: a plausible explanation for infertility. Microb Pathog.

[15] Hassani S, Asghari G, Yousefi H (2013). Effects of different extracts of eucalyptus camaldulensis on trichomonas vaginalis parasite in culture medium. Adv Biomed Res.

[16] Hernández Ceruelos A, Romero-Quezada LC, Ruvalcaba Ledezma JC (2019). Therapeutic uses of metronidazole and its side effects: an update. Eur Rev Med Pharmacol Sci.

[17] Boskabady MH, Shafei MN, Saberi Z (2011). Pharmacological effects of rosa damascena. Iran J Basic Med Sci.

[18] Committee HMD (2001). Iranain herbal pharmacopea.

[19] Khazaeli P, Mehrabani M, Mosadegh A (2020). Formulation, physiochemical, and microbial assay of henna oil vaginal suppository formulated with polyethylene glycol bases. Iran J Med Sci.

[20] Raju GS, Moghal MR, Dewan SMR (2013). Characterization of phytoconstituents and evaluation of total phenolic content, anthelmintic, and antimicrobial activities of solanum violaceum ortega. Avicenna J Phytomed.

[21] Khan W, Subhan S, Shams DF, et al. Antioxidant potential, phytochemicals composition, and metal contents of datura alba. Biomed Res Int. 2019:2403718. 10.1155/2019/2403718. 10.1155/2019/2403718 PMC6601491 31317024

[22] Requena R, Vargas M, Chiralt A. Study of the potential synergistic antibacterial activity of essential oil components using the thiazolyl blue tetrazolium bromide (Mtt) Assay. Lwt. 2019;101(183-90).

[23] Barrak I, Baráth Z, Tián T, et al. Effects of different decontaminating solutions used for the treatment of peri-implantitis on the growth of porphyromonas gingivalis-an in vitro study. Acta Microbiol Immunol Hung. 2020;68(1):40–7. 10.1556/030.2020.01176. 10.1556/030.2020.01176 32845853

[24] Johnson TL, Forbes BA, O’Connor-Scarlet M (1985). Rapid method of mic determinations utilizing tetrazolium reduction. Am J Clin Pathol.

[25] Alizadeh Behbahani B, Shahidi F (2019). Melissa officinalis essential oil: chemical compositions, antioxidant potential, total phenolic content and antimicrobial activity. Nutr Food Sci Res.

[26] Mirzaei F, Bafghi AF, Mohaghegh MA (2016). In Vitro anti-leishmanial activity of satureja hortensis and artemisia dracunculus extracts on leishmania major promastigotes. J Parasit Dis.

[27] Sáez-López E, Guiral E, Fernández-Orth D (2016). Vaginal versus obstetric infection escherichia coli isolates among pregnant women: antimicrobial resistance and genetic virulence profile. PLoS One.

[28] Hirulkar N, Agrawal M (2010). antimicrobial activity of rose petals extract against some pathogenic bacteria. Int J Pharm Biol Arch.

[29] Štumpf S, Hostnik G, Primožič M (2020). The effect of growth medium strength on minimum inhibitory concentrations of tannins and tannin extracts against E. Coli. Molecules.

[30] TingWu, He M, Zang X (2031). A structure–activity relationship study of flavonoids as inhibitors of E. Coli by membrane interaction effect. Biochim Biophys Acta Biomembr.

[31] Rosen GH, Randis TM, Desai PV (2017). Group B Streptococcus and the vaginal microbiota. J Infect Dis.

[32] Mahboubi M, Kazempour N, Khamechian T (2011). Chemical composition and antimicrobial activity of rosa damascena mill essential oil. J Biol Active Prod Nat.

[33] Halawani EM (2014). Antimicrobial activity of rosa damascena petals extracts and chemical composition by gas chromatography-mass spectrometry (Gc/Ms) analysis. Afr J Microbiol Res.

[34] Nayebi N, Khalili N, Kamalinejad M (2017). A systematic review of the efficacy and safety of rosa damascena mill. With an overview on its phytopharmacological properties. Complement Ther Med.

[35] Behzadi P, Baráth Z, Gajdács M. It’s not easy being green: a narrative review on the microbiology, virulence and therapeutic prospects of multidrug-resistant pseudomonas aeruginosa. Antibiotics. 2021;10(1). 10.3390/antibiotics10010042. 10.3390/antibiotics10010042 PMC7823828 33406652

[36] Trong Le N, Viet Ho D, Quoc Doan T (2020). Biological activities of essential oils from leaves of paramignya trimera (Oliv.) guillaum and limnocitrus littoralis (Miq.) Swingle. Antibiotics.

[37] Shohayeb M, Abdel-Hameed E-SS, Bazaid SA (2014). Antibacterial and antifungal activity of rosa damascena mill. Essential oil, different extracts of rose petals. Glob J Pharmacol.

[38] Thomas NV. Control of opportunistic bacteria using aqueous and ethanol extracts of rosa damascena. Int J Microbiol Res. 2020;12(2):1176-8.

[39] Abu-Shanab B, Adwan GM, Jarrar N (2007). Antibacterial activity of four plant extracts used in palestine in folkloric medicine against methicillin-resistant Staphylococcus Aureus. Turk J Biol.

[40] Mankar S (2015). Screening of antibacterial activity of rose varieties against bacterial pathogens. Int J Life Sci.

[41] Kashani LM-T, Memarzadeh MR, Hatami A (2016). Comparison of two different traditional methods of rose oil preparation in terms of physicochemical factors. Trad Integr Med.

[42] Ortiz-de la Tabla V, Gutiérrez F (2019). Cervicitis: etiología, diagnóstico Y tratamiento. Enferm Infecc Microbiol Clin.

[43] Rakhshandah H, Shakeri MT, Ghasemzadeh MR (2010). Comparative hypnotic effect of rosa damascena fractions and diazepam in mice. Iran J Pharm Res.

[44] Hajhashemi V, Ghannadi A, Hajiloo M (2010). Analgesic and anti-inflammatory effects of rosa damascena hydroalcoholic extract and its essential oil in animal models. Iran J Pharm Res.

[45] Latifi G, Ghannadi A, Minaiyan M (2015). Anti-inflammatory effect of volatile oil and hydroalcoholic extract of rosa damascena mill. On acetic acid-induced colitis in rats. Res Pharm Sci.

[46] Trong Le N, Viet Ho D, Quoc Doan T, et al. In Vitro antimicrobial activity of essential oil extracted from leaves of leoheo domatiophorus chaowasku, D.T. Ngo and H.T. Le in Vietnam. Plants. 2020;9(4). 10.3390/plants9040453. 10.3390/plants9040453 PMC7237999 32260297

[47] Ezatpour B, Badparva E, Ahmadi S (2009). Investigation of anti trichomonas vaginalis activity of lavandula angyustifolia essential oil in invitro media. SJIMU..

[48] Alyasari HF, Al-khafaji JK, Al-Masoudi HK (2018). Inhibitory effects of garlic extract on uropathogenic escherichia coli; proteus mirabilis and trichomonas vaginalis isolated from urogenital tract cases. Res J Pharm Technol.

[49] Bala V, Chhonker YS (2018). Recent developments in anti-trichomonas research: an update review. Eur J Med Chem.

[50] Silva BM, Andrade PB, Valentão P (2004). Quince (Cydonia Oblonga Miller) Fruit (Pulp, Peel, and Seed) and Jam: antioxidant activity. J Agric Food Chem.

